# The Use of Natural Biopolymer of Chitosan as Biodegradable Beads for Local Antibiotic Delivery: Release Studies

**Published:** 2013-02-13

**Authors:** Jebraeel Movaffagh, Ali Ghodsi, BiBi Sedigheh Fazly Bazzaz, Sayyed Abolghassem Sajadi Tabassi, Hamideh Ghodrati Azadi

**Affiliations:** 1 Targeted Drug Delivery Research Center, Department of Pharmaceutics, School of Pharmacy, Mashhad University of Medical Sciences, Mashhad, IR Iran; 2 Biotechnology Research Center, School of Pharmacy, Mashhad University of Medical Sciences, Mashhad, IR Iran; 3 Pharmacological Research Center of Medicinal Plants, School of Medicine, Mashhad University of Medical Sciences, Mashhad, IR Iran; 4 Department of Basic Sciences, Fcaulty of Veterinary Medicine, Ferdowsi University of Mashhad, Mashhad, IR Iran

**Keywords:** Delayed-action Preparations, Teicoplanin, Chitosan, Cross-Linking Reagents

## Abstract

**Background:**

Chitosan is a naturally occurring biopolymer which has been widely used in a variety of biomedical applications including local antibiotic delivery due to its excellent mechanical properties, biodegradability and biocompatibility. Beads are spherical, porous carriers which are prepared from various materials including chitosan.

**Objectives:**

The current study aimed to fabricate a new controlled delivery system for local anti-infective treatment and to study its release behavior.

**Materials and Methods:**

Twenty beads were prepared from 1% or 2% chitosan solutions and immersed in vancomycin (VM) or teicoplanin (TN) solutions. The antibiotic release kinetics was determined by linear regression analysis supposing first order kinetics.

**Results:**

Immersion for 3 h resulted in significant increase in the total TN release that differed from 0.5 h of immersion, except for the 1% beads immersed in VM. Increasing the chitosan concentration significantly increased the total release and antibiotic load of beads. The release of TN was more delayed compared to that of VM, which allowed a gradual release beyond 3 days. The half-life (mean ± SEM) of both types of TN-containing beads was significantly extended for 3 h immersion in comparison to 0.5 h immersion (26.1 ± 5.9 vs 10.9 ± 1.0 and 17.0 ± 2.1 vs 5.1 ± 1.9; P < 0.001). However, neither increasing the chitosan concentration, nor immersion time did result in any significant increase in the release of VM.

**Conclusions:**

The current study demonstrated an improved control of TN release impregnated in beads. It can be concluded that chitosan beads might be considered as a novel carrier for TN delivery to infected bone for local anti-infective therapy.

## 1. Background

**Figure 1:**
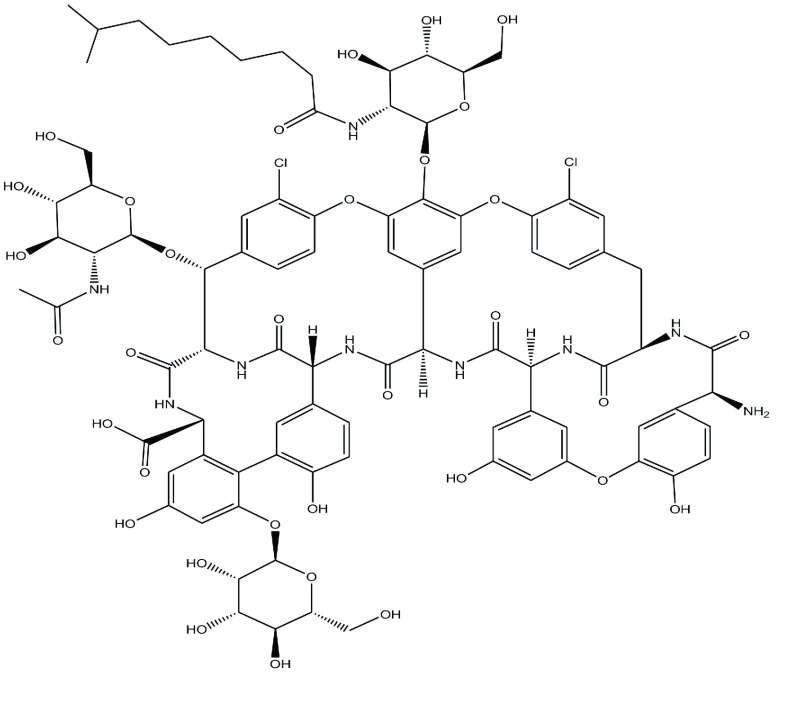
Chemical Structure of Teicoplanin as a Typical Lipoglycopeptide Antibiotic

In spite of various viewpoints and concepts about the efficacy of biodegradable antibiotic beads, there is an increasing acceptance for sustained antibiotic release systems in surgical practice. After development of polymethylmethacrylate (PMMA) bone cement containing gentamicin by Bucholz and Engelbrecht, Klemm developed PMMA gentamicin chains, which are still in use together with the surgical treatment of bone and soft tissue infections today (1, 2). Chitosan with excellent biodegradable and biocompatible characteristics, is a naturally occurring polysaccharide. Chitosan has been extensively studied as a carrier for drugs due to its unique polymeric cationic character and its gel and film forming properties. Chitosan has been examined extensively in the pharmaceutical industry for its potential in the development of drug delivery systems (3). Cross-linked chitosan beads not only are insoluble in acid solution but also present higher surface areas and stronger mechanical properties than the raw chitosan powder and other adsorbents (4). Later on, biodegradable antibiotic delivery systems, mainly containing aminoglycosides, were introduced to clinical therapy. During the last 30 years, infections due to methicillin-resistant staphylococcus aureus (MRSA) have become a problem (5). For these infections, lipoglycopeptide antibiotics such as vancomycin (VM) and teicoplanin (TN) have proven to be the most efficient with known low resistance rates (6). TN at the viewpoint of chemical properties, has three pKa values (5.0, 7.1 and 9.2) and is soluble in water (1:10) but has very low solubility in alcohol (1:1000) and has three sugar moieties in its formula which could interact with polysaccharide chains of chitosan. VN has similar structure to TN and is used as hydrochloride which is freely soluble in water. There is a significant difference in their structures naming; TN has a different pattern of glycosylation, and contains an N-decanoyl side chain attached through 2-aminoglucose which render its interaction with polymer chain more severely. Because of detecting MRSA which is a Gram-positive bacteria in most cases of osteomyelitis and bone infections, and also the effectiveness of VM against most Gram-positive bacteria, this drug was selected. TN interferes with the final stage of peptidoglycan biosynthesis. The antibacterial spectrum of TN is similar to that of VM and encompasses most species of important Gram-positive pathogens (Figure 1). The core aglycone is a linear heptapeptide of seven aromatic amino acids with chlorine atom found on each of the tyrosine (Tyr) residues. Three sugar moieties are attached to the aryl groups: α-D-mannose at amino acid 7, N-acetyl-β-D-glucosamine at amino acid 6, and N-fatty acyl-β-D-glucosamine at amino acid 5. The acyl moiety is a fatty acid residue containing 9-12 carbon atoms.

TN is two- to four-fold more active than VM against both methicillin-susceptible and methicillin-resistant isolates of S. aureus with minimum inhibitory concentration (MIC90) typically in the range of 0.51 and 1-2 mg/L, respectively (7). Chitosan [β (1, 4) 2-amino-2-D-glucose] is a cationic biopolymer, produced by alkaline N-deacetylation of chitin, which is the constituting element of the shells of crustaceans such as crabs and shrimps (8, 9). Chitosan is a functional linear polymer which is the most abundant natural polysaccharide on the earth after cellulose. Chitosan is a biocompatible and biodegradable element with healing and haemostatic properties and is extensively used in tissue engineering and as a drug carrier system (10). The preparation of chitosan beads and microgranules comes form the basis of a report on chitosan as a drug carrier for disodium diclofenac (11-13). Following excellent properties of chitin and chitosan for adsorbing elements Onishi et al. prepared microspheres by drawing on the complex coacervation ability of Fe (III) ions with 6-O-carboxymethyl-chitin (8, 14).

## 2. Objectives

The present study aimed to prepare chitosan beads loaded with VM and TN and investigate the release profiles of the loaded drugs from the beads intended to be used in the treatment of osteomyelitis.

## 3. Materials and Methods

### 3. 1. Materials

Vancomycin hydrochloride (Zakaria-Tabriz Pharmaceutical Co, Tabriz, Iran) and teicoplanin as Targocid® (Sanofi Aventis pharmaceutical Ltd., Swiss) were purchased from pharmacies. Chitosan with medium molecular weight and 85% degree of deacetylation (DD) (Aldrich 448877) Glutaraldehyde 50% (Molekula, Dorset SP8 4PX U K), Mueller-Hinton Broth, Mueller-Hinton agar and nutrient agar were purchased from HiMedia Laboratories (Mumbai, India). All other chemicals were of reagent grade unless otherwise stated and used as received.

### 3. 2. Preparation of Chitosan Beads

Chitosan (1 and 2% w/v) was dissolved in an aqueous solution of 1% (w/v) acetic acid under gentle stirring (45 rpm) for 14-16 hours at room temperature. Ten grams of polymer solution was cross-linked with 0.1 mL glutaraldehyde diluted in PBS (GTA, 25%w/w) and dropped into a liquid sodium hydroxide 1.5 M through injector (15). Stock solutions of vancomycin hydrochloride and teicoplanin were prepared according to the manufacturer’s guidelines at a concentration of 20 mg/mL for vancomycin and 8 mg/mL for teicoplanin and stored at -20°C until used. Loading of vancomycin and teicoplanin in chitosan beads was performed after bead preparation. After 15 min of crosslinking and insolubilization, the drug unloaded beads were filtered by a paper filter and rinsed in distilled water at 25°C to eliminate the residual of unreacted GTA and extra NaOH completely (14, 15). In immersion method, the newly prepared swollen chitosan beads were immersed in 50 mL of antibiotic solution under gentle stirring at 25°C. All procedures were performed in darkness. Beads were freeze dried for future works. The different series of investigated formulations are listed in (Table 1). The release of antibiotic by diffusion from 20 antibiotic loaded chitosan beads was investigated by elution in 100 mL of PBS at pH 7.4 and 37°C for 7 days. Samples were taken at 1, 2, 3, 4, 5, 6, 8, 12, 18, 24, 36, 48, 60 and 96 h of elution. The amount of drug release at each sampling time was measured based on the concentrations. It is also valuable to mention that for each sampling 4 mL of release medium was taken and replaced with fresh medium according to the given schedule. Although the processes of diffusion are theoretically infinite, the final sampling was taken after 96 h. The final release of both antibiotics to this point was supposed to be practicality 100%.

**Table 1:** Different Series of Bead Formulations Investigated

Series Name	Type of Antibiotic	Chitosan Concentration, g/100	Time of immersion, h
**VM 0.5 h 1%**	VM	1	0.5
**VM 0.5 h 2%**	VM	2	0.5
**VM 3 h 1%**	VM	1	3
**VM 3 h 2%**	VM	2	3
**TN 0.5 h 1%**	TN	1	0.5
**TN 0.5 h 2%**	TN	2	0.5
**TN 3 h 1%**	TN	1	3

Abbreviations: VM,Vancomycin; TN,Teicoplanin; 1%/2%, chitosan concentrations (w/v); 0.5h/3h, time of immersion in antibiotic solution.

### 3. 3. Release Kinetics and Half-life (t _1/2_)

To investigate whether drug release profiles follow zero-order kinetic, ([D]/ [D]_0_) was plotted against time. Giving a straight line function with slope -k would show zero order release kinetic, the equation of first-order release will become as: 

(d [D]) / [D] = - kdt

Where D is the concentration of the drug and k is the release rate constant. That can be integrated simply, because k is a constant and independent of t, and become:

[D]=[D]_0_ e^-kt^

Where [D] _0_ is the initial concentration of drug and [D] is the concentration at any time point. This means that the ratio of concentrations ([D] / [D]_0_ ) declines exponentially as a function of time. The rate constant k in a first order reaction which can also be determined from the t _1/2_. To calculate the half-life t_1/2_, the equation of first-order release can also be written as:

Kt_1/2_ =( ln ([D]_0_ /( [D]_0_ /2))). Hencet_1/2_ = ln (2/k)

The release rate constant k was computed for each test by calculating the slope of the line for LN [D] plotted against time.

### 3. 4. Bioassay of Elutes

To monitor any potential, chemical, or physical interference of the drug molecules with the chitosan beads, 30 random elution samples were again tested by agar diffusion method according to NCCLS guidelines (16, 17). The reference microorganism for vancomycin and teicoplanin was Bacillus subtilis ATCC 6633 (18). A total of 50 mL of sterile nutrient agar (HiMedia Lab., Mumbai, India) was seeded with the stated bacteria at a concentration of 108 cfu/l and poured into round Petri dishes of 22 cm diameter. Then, 14 cylinders were placed onto the agar and filled with 200 μl of the elution samples and antibiotic calibrators. Calibrator concentrations of vancomycin and TN were 6.25, 12.5, 25, and 50 mg/l, prepared in PBS. After Incubation at 37°C for 18 h, the inhibition zones were analyzed. The antibiotic concentration of the elutes was determined by regression analysis.

### 3. 5. Statistical Analysis

All data were analyzed using the SPSS software (Version 20.00). Results were expressed as mean values ± standard error of the mean (SEM). Values of P < 0.05 were considered significant. Data of all groups were proven to adhere to a standard normal distribution. Differences between groups were tested by an analysis of variences (ANOVA), followed by a post hoc comparison using the Newman-Keuls method. Correlation between the agar diffusion microbiological assay and the spectrophotometrical assay was assessed using the correlation coefficient according to Spearman.

## 4. Results

**Figure 2:**
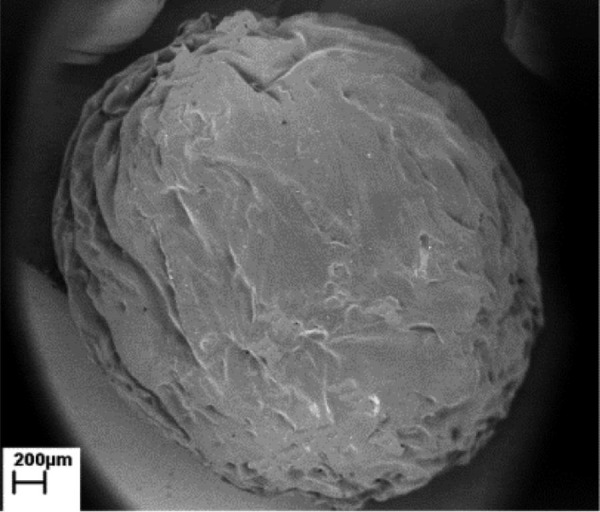
The Morphology of a Single Spherical Bead

**Figure 3:**
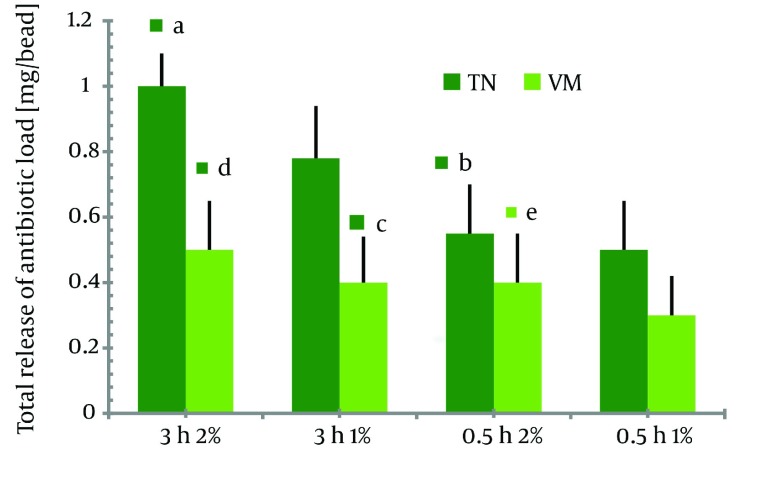
Total Release of VM and TN After 96-h Elution From Chitosan Beads. Data are Presented According to Bead Formulations of Varying Chitosan Concentrations and Immersion Time (^a^P < 0.001 vs series TN 0.5h 2%; ^b^P < 0.05 vs series TN 0.5h 1%; ^c^P < 0.05 vs series Tn 3h 1%; ^d^P < 0.05 vs series VM 0.5h 2%; ^e^P > 0.05 vs series VM 3h 1%; and P < 0.001 vs series TN 0.5h 2%)

The mean weight of the twenty beads with the same dimensions prepared from 1% and 2% chitosan was 150.3 mg ± 57.1 and 200.1 mg ± 63.3, respectively. The surface and morphology of a single bead is illustrated in ( Figure. 2 ). As shown in ( Figure 3 ), prolonging the immersion time in antibiotic solution from 0.5 h to 3 h was accompanied with a significant increase in total drug load for both types of TN-containing beads (P < 0.001). However, for vancomycin the same effect was shown only for the 2%-beads and not for the 1%-beads (P < 0.05 and P > 0.05). In addition, the total release of TN significantly increased by using 2%-beads in comparison to that of 1%-beads (P < 0.001). However, this effect was only shown for beads that had been subjected to a 3 h immersion period in antibiotic solution.

**Figure 4:**
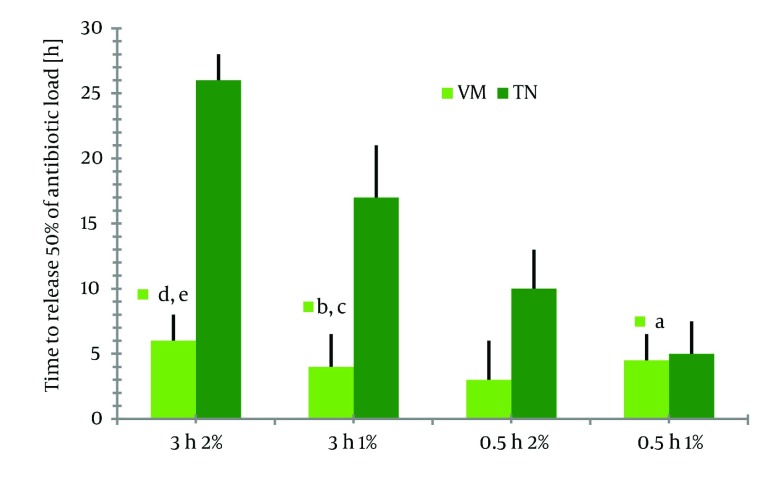
Time for Beads to Release 50% of Total Antibiotic Load (t_1/2_: half-life). Values are Given in Hour. (^a^P > 0.05 vs series VM 0.5h 2%; ^b^P > 0.05 vs series VM 0.5h 1%; ^c^P < 0.05 vs series VM 3h 1%; ^d^P < 0.001 vs series TN 0.5h 2%; ^e^P < 0.001 vs series VM 3h 2%)

**Figure 5:**
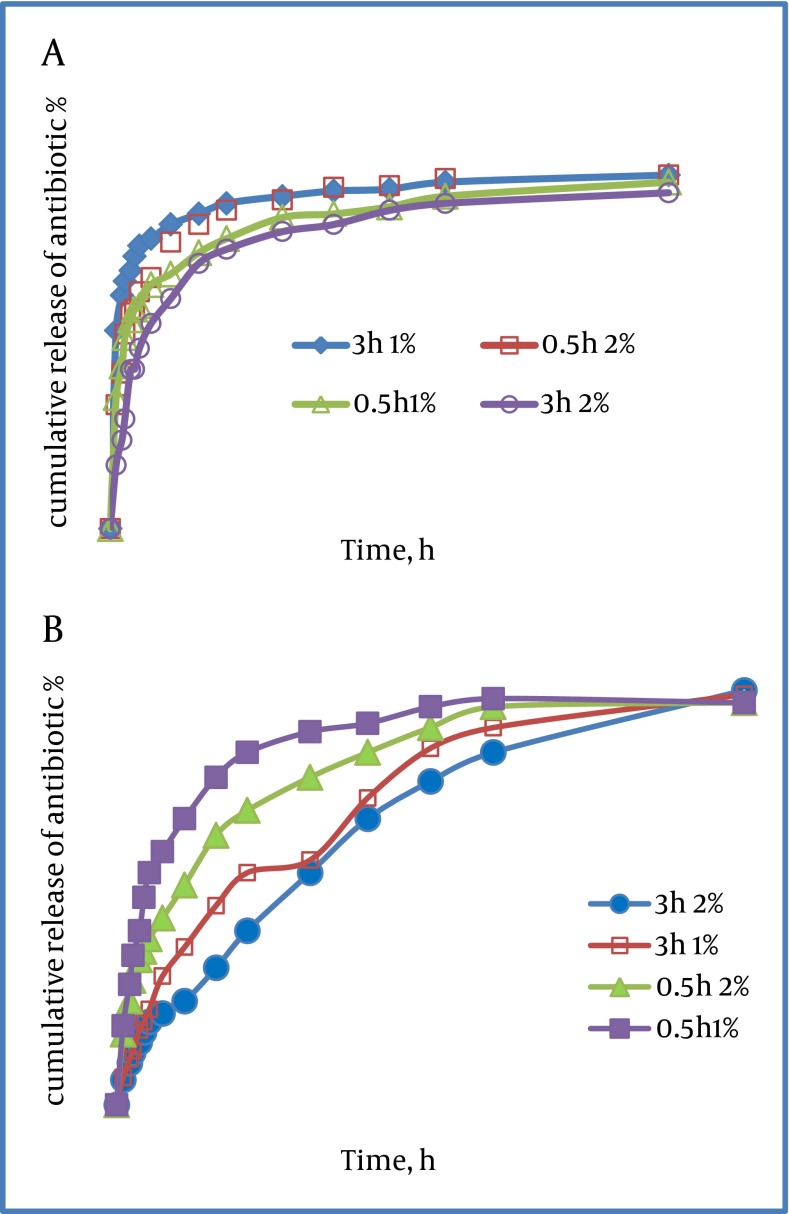
Release of (a) VM and (b) Teicoplanin From Chitosan Beads by Different Chitosan Solutions and After 0.5 h and 3 h of Immersing in Antibiotic Solution. Values Given in Percentages of Total Antibiotic Release With SD.

Increasing the chitosan concentration for the beads had negative effect upon total TN and VM release (P < 0.05). This was shown by a significant decrease in antibiotic release in both series of 2%-beads compared to corresponding series of 1%-beads. After each experiment, tests were conducted to determine whether the hypothesis of first-order release rate was true, and the half-life was calculated according to the above procedure. Antibiotic release profiles and t1/2 of all different series are illustrated in Figures 3 and 4 respectively. As shown in Figure 5 (a,b) the release of TN was found to be more prolonged than that of VM. This was confirmed by the significantly longer period of time needed to reach the 50% release of the total antibiotic content, compared to corresponding series of VM loaded beads.

**Figure 6:**
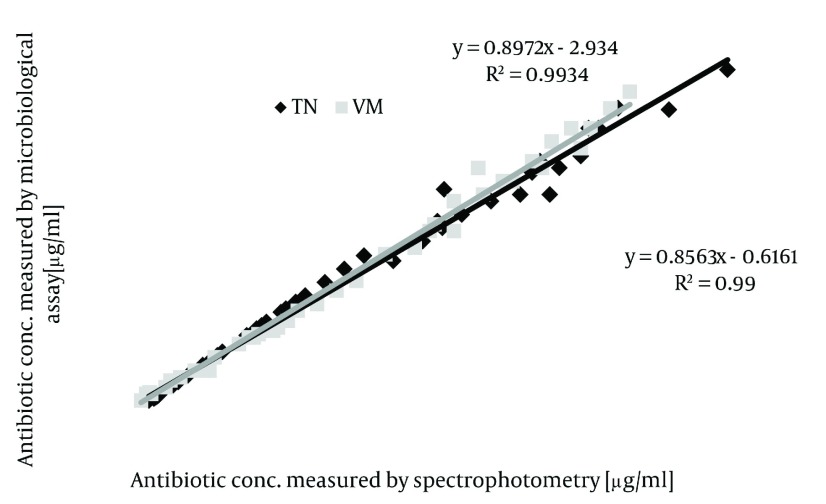
The Correlation of Concentrations of VM and TN After Elution From Chitosan Beads in PBS pH 7.4, Assessed by two Methods of Measurement.

For the series of VM 0.5 h 1%, the differences in half-life between both antibiotics did not show significant difference (series VM 0.5 h 1% vs series TN 0.5 h 1%; P>0.05 as determined by ANOVA). In comparison to all other investigated series, drug release was fast in 1%-chitosan beads immersed in VM solution for 3h (for the series of VM 0.5h, 1%). Here, more than 60% of total antibiotic load was released within 3 h. Elution was nearly complete (95%) after 36 h Figure. 5a . In contrast, release of TN from 1% and 2% beads after 3 h immersion (series TN 3h 1% and series 3h 2%) followed a nearly zero-order release rate, indicating a sustained delivery even after 3 days Figure 5a . This was achieved by a prolonged immersion in TN solution for both types of beads (series TN 3h 1% vs series TN 0.5h 1%; P < 0.001, series TN 3 h 2% vs series TN 0.5 h 2%; P < 0.001 as determined by ANOVA). In the case of VM, however, either increasing the chitosan content of the beads or an extended immersion time could yield a significantly prolonged release. The release of TN was, in general, significantly prolonged compared to corresponding series of VM. The data for release kinetics were illustrated in ( Table 2 ). The mechanism of drug diffusion deviates from the Fickian equation Values. The amount below 0.43 indicate that drug release from polymer was due to Fickian diffusion (19, 20). The results of microbiological assay (plotted on the y axis) and the spectrophotometric assay (plotted on the x axis) showed the same levels of lipoglycopeptide concentrations in all samples tested as indicated in ( Figure 6 ). To be confident about the absence of any interaction between antibiotics and chitosan, DSC procedures were conducted from 500-4000cm-1. As a result of the Tg and melting points shown on thermograms (data not presented) of polymer and drugs no interactions were observed between drugs and polymer. Using the correlation coefficient, we had a good level of agreement between concentrations measured by spectrophotometric bioassay methods with a correlation coefficient of r2 > 0.95 (P < 0.001) according to Spearman statistical test. It can be assumed that the presence of loaded antibiotics within chitosan beads does not alter the antimicrobial properties of neither lipoglycopeptide antibiotics.

**Table 2:** Release Kinetics of Different Bead Formulations With Three Replicates (P < 0.05)

Different Bead Formulations	K	N	R
**VM 0.5 h 1%**	56.0	0.30	0.96
**VM 0.5 h 2%**	51.0	0.26	0.95
**VM 3 h 1%**	61.0	0.31	0.97
**VM 3 h 2%**	45.0	0.35	0.97
**TN 0.5 h 1%**	10.1	0.42	0.98
**TN 0.5 h 2%**	10.0	0.41	0.97
**TN 3 h 1%**	10.2	0.40	0.96
**TN 3 h 2%**	8.9	0.41	0.98

## 5. Discussion

Antibiotic delivery systems for local anti-infective treatment are frequently used in nearly all cases of surgery, especially in cases of chronic osteomyelitis and deep tissue infection. The major advantage of local application of antibiotics is producing high concentration in the infected sites without toxic effects due to systemic administration. Biodegradable antibiotic delivery systems are proven to have advantages over inert polymers (11, 21, 22). Once they are implanted, there is no need for a second surgical procedure for removal. In addition, they release their entire antibiotic load because of their complete biodegradability. This opposes the limited portion released by nondegradable materials such as PMMA gentamicin beads. As reported earlier loading antibiotics in chitosan could provide alternative methods to treat musculoskeletal infections and chitosan could enhance bone reconstruction and regeneration. The current study used medium molecular weight chitosan formulation. Since Staphylococcus aureus is still the predominating organism in nearly all types of surgical wound infections, and because of increasing resistances to methicillin and gentamicin, the researchers used these therapeutics systems in the current investigation. In spite of local pain reports and tissue necrosis due to intravenous injection, some in vivo studies have reported the effectiveness of a local antibiotic delivery using VM, indicating a low antigenicity and little toxic effects (11, 23). Accordingly, studies performed on the cytotoxicity of VM and TN in cultured human keratinocytes and fibroblasts showed a high level of compatibility (24). However, a significantly higher grade of morphological cell damage and a higher LDH release in endothelial cell cultures were seen for VM versus TN. This may point to higher therapeutic safety for TN in local drug delivery (21). The current study data showed that the overall sustained release characteristics of TN were higher than those of VM. In all series of VM containing beads, 50% of antibiotic load was released before 45 h of elution, regardless of the chitosan concentration and the immersion period. VM release rates were significantly higher in all corresponding series except VM 0.5 h 1%. In release profiles of TN loaded beads the fastest antibiotic delivery of about 60% of total load within the 40th hour was observed for 1% chitosan bead immersed in TN solution for 0.5 h (series TN 0.5 h 1%). The results shown in Table 2 indicate that the drug release mechanism is Fickian for dry chitosan beads. The current study results suggest that the longer durability of TN was achieved by the chitosan carrier system used in this study. Difference between release rates of VM and TN in the current study may be due to the higher electrostatic and hydrogen binding of TN and due to its more lipophilic groups in comparison to the weak value stated for VM (25). However, the underlying mechanism of retention of TN within the bead remains unclear and requires further investigation. In addition, this study discovered a significant increase in total antibiotic release for both drugs using an expanded immersion time of 3 h instead of 0.5 h, except for the 1%-beads immersed in TN. This observation supports the theory that extended immersing time may be related to a better saturation and of the bead with antibiotic solution and higher adsorption during the immersion process. So, the current study results of the bioassay verify that the antimicrobial properties of both antibiotics were not altered by interaction with the chitosan bead even after extended periods of immersion. Chitosan beads could be considered as a promising carrier to apply TN and/or VM to infected sites for local anti-infective therapy. Preference should be given to TN-containing chitosan beads because of their more linear release characteristics. Nevertheless, follow-up tests, simulating the conditions involved in infective tissue, are necessary to verify definite therapeutic efficacy.
